# Sway‐dependent changes in standing ankle stiffness caused by muscle thixotropy

**DOI:** 10.1113/JP271137

**Published:** 2015-12-30

**Authors:** Tania E. Sakanaka, Martin Lakie, Raymond F. Reynolds

**Affiliations:** ^1^ School of Sport, Exercise & Rehabilitation SciencesUniversity of BirminghamBirminghamUK

## Abstract

**Key points:**

- The passive stiffness of the calf muscles contributes to standing balance, although the properties of muscle tissue are highly labile.
- We investigated the effect of sway history upon intrinsic ankle stiffness and demonstrated reductions in stiffness of up to 43% during conditions of increased baseline sway.
- This sway dependence was most apparent when using low amplitude stiffness‐measuring perturbations, and the short‐range stiffness component was smaller during periods of high sway.
- These characteristics are consistent with the thixotropic properties of the calf muscles causing the observed changes in ankle stiffness.
- Periods of increased sway impair the passive stabilization of standing, demanding more active neural control of balance.

**Abstract:**

Quiet standing is achieved through a combination of active and passive mechanisms, consisting of neural control and intrinsic mechanical stiffness of the ankle joint, respectively. The mechanical stiffness is partly determined by the calf muscles. However, the viscoelastic properties of muscle are highly labile, exhibiting a strong dependence on movement history. By measuring the effect of sway history upon ankle stiffness, the present study determines whether this lability has consequences for the passive stabilization of human standing. Ten subjects stood quietly on a rotating platform whose axis was collinear with the ankle joint. Ankle sway was increased by slowly tilting this platform in a random fashion, or decreased by fixing the body to a board. Ankle stiffness was measured by using the same platform to simultaneously apply small, brief perturbations (<0.6 deg; 140 ms) at the same time as the resulting torque response was recorded. The results show that increasing sway reduces ankle stiffness by up to 43% compared to the body‐fixed condition. Normal quiet stance was associated with intermediate values. The effect was most apparent when using smaller perturbation amplitudes to measure stiffness (0.1 *vs*. 0.6 deg). Furthermore, torque responses exhibited a biphasic pattern, consisting of an initial steep rise followed by a shallower increase. This transition occurred earlier during increased levels of ankle sway. These results are consistent with a movement‐dependent change in passive ankle stiffness caused by thixotropic properties of the calf muscle. The consequence is to place increased reliance upon active neural control during times when increased sway renders ankle stiffness low.

## Key points

- The passive stiffness of the calf muscles contributes to standing balance, although the properties of muscle tissue are highly labile.
- We investigated the effect of sway history upon intrinsic ankle stiffness and demonstrated reductions in stiffness of up to 43% during conditions of increased baseline sway.
- This sway dependence was most apparent when using low amplitude stiffness‐measuring perturbations, and the short‐range stiffness component was smaller during periods of high sway.
- These characteristics are consistent with the thixotropic properties of the calf muscles causing the observed changes in ankle stiffness.
- Periods of increased sway impair the passive stabilization of standing, demanding more active neural control of balance.

**Abbreviations**
COM: centre of mass
EMG: electromyogram
SREC: short‐range elastic component

## Introduction

In quiet standing, the body's centre of mass is situated forward of the ankle joint, and so continuous ankle torque is required to prevent it from falling forwards (Schieppati *et al*. 1994; Gatev *et al*. 1999). This torque arises from two sources: active and passive. Here, the passive mechanism refers to the natural viscoelastic resistance of the ankle joint to forward body motion, assuming a fixed level of muscle activity. It does not imply that the musculature is relaxed but, instead, that the level of activity is not altered by the nervous system. Conversely, the active mechanism is the modulation of the calf muscle activity by the nervous system. Previous research has confirmed that the passive stiffness of the ankle joint alone is insufficient to stabilize the body (Morasso & Schieppati, 1999; Loram & Lakie, 2002; Morasso & Sanguineti, 2002). This low stiffness is largely a result of the high compliance of the long Achilles tendon exposed to the relatively low ankle torque involved in quiet stance. Therefore, the passive mechanism must be supplemented by the active mechanism. However, their relative importance differs considerably, both between and within individuals. Between‐subject differences are indicated by the considerable variation in intrinsic ankle stiffness measured using rotary perturbations (Loram & Lakie, 2002; Casadio *et al*. 2005). This has important implications for the neural control of balance because individuals who have inherently stiffer ankle joints (e.g. as a result of a stiffer Achilles tendon) can rely more upon the passive mechanism and less upon active modulation. Within‐subject differences were identified in studies in which human joints were perturbed in various ways (Halaki *et al*. 2006; Loram *et al*. 2007 *a*). To our knowledge, however, the source and significance of these differences has not been clarified fully. In the present study, we investigate these within‐subject differences.

How might short‐term changes in intrinsic ankle stiffness occur within a person? Two of the main contributory structures to passive ankle stiffness during stance are the Achilles tendon and the triceps surae muscles. In quiet standing, where the stretch sizes are normally very small, the muscle is typically ∼15 times stiffer than the tendon (Loram *et al*. 2007 *b*; Loram *et al*. 2009). Because the two structures are arranged in series, the limiting factor in overall ankle stiffness is therefore normally the tendon. This assumes no significant changes in stiffness over time. However, although tendon stiffness changes relatively slightly and slowly, the mechanical properties of muscle tissue are highly labile. When a relaxed muscle fibre is stretched or shortened, there is an initial period of relatively high resistance, termed the short‐range elastic component (SREC) (Hill, 1968). This phenomenon is dependent on two factors: displacement amplitude and history of movement. After a position threshold is reached, resistance to movement drops markedly and the initial high stiffness of the SREC disappears. This effect is greatly reduced when the muscle is stretched immediately after a prior stretch, with the initial SREC becoming much smaller. The stiffness of the SREC gradually recovers but only if the muscle is left still over a period of seconds. This temporary reduction in muscle stiffness caused by movement, with recovery at rest, is known as muscle thixotropy (Denny‐Brown, 1929; Hill, 1968; Lakie & Robson, 1990; Warner & Wiegner, 1990; Whitehead *et al*. 2001). These two effects are considered to be a result of forced detachment and spontaneous reattachment of some muscle cross‐bridges over time in relaxed muscle (Hill, 1968; Campbell & Lakie, 1998; Altman *et al*. 2015). Various *in vivo* experiments have also detected these patterns at the initial stages of movement in muscle where at least part of it is tonically active. This is the manifestation of the observations of Hill (1968) with respect to amphibian muscle fibres. Large limb movements encounter less stiffness than small ones over a range of background muscle activations (Rack & Westbury, 1974; Halaki *et al*. 2006). Moreover, after large joint limb movements, this reduction in stiffness persists for a short time, recovering rapidly if the system is left still (Lakie *et al*. 1984; Proske *et al*. 1993; Axelson & Hagbarth, 2001; Reynolds & Lakie, 2010).

This raises the likelihood that the intrinsic ankle stiffness in standing individuals, which is highly dependent on the muscle properties, is also affected by the transient characteristics of its short‐range stiffness. Loram *et al*. (2007*a*) previously investigated the effect of amplitude in standing individuals and reported that ankle stiffness is indeed less for larger movements (see also Kearney & Hunter, 1982; Vlutters *et al*. 2015). To our knowledge, however, the effects of thixotropy in maintaining posture are yet to be investigated. We speculated that intrinsic ankle stiffness would be greater when measured with small perturbations only when the system was moving minimally, sway size was small, and there was an opportunity for stiffness recovery. When there is an increased amount of baseline body sway, there would be negligible recovery of stiffness and ankle stiffness would be less for all sizes of perturbation. In the present study, we test this hypothesis by manipulating sway size, or ankle motion, in three standing conditions. First, we study normal quiet stance. Then, we use a rotating platform, whose axis is collinear with the ankle joint, to increase sway size. Lastly, we strap the body to a stationary backboard to minimize sway. Passive intrinsic ankle stiffness is measured in all three situations by applying small (<0.6 deg) and brief (<140 ms) perturbations using the rotating platform. In addition to changing the history of movement to measure the thixotropic aspect, we also change the amplitude of stimuli to assess stiffness, over the range 0.1–0.6 deg. By clarifying whether the intrinsic stiffness of the ankles is simultaneously dependent on these two independent factors, we can then confirm that the changes within subjects, in quiet standing, are a result of the passive mechanical properties specific to the short‐range stiffness of the muscle. The implication of an ankle stiffness that depends on the history of movement is that the demand for neural intervention to stabilize standing will not be constant but, instead, will vary continuously. It will be minor when sway size is small and intrinsic stiffness is high. By contrast, it will be disproportionately greater when there is a history of large sway size and intrinsic stiffness is reduced. This means that the minimization of neural effort is assured by keeping sway size small. Conversely, large sways can produce a decrease in stability and will require considerable neural intervention (Sozzi *et al*. 2013).

## Methods

### Participants

Ten healthy subjects (two female, eight male; mean ± SD age 30.9 ± 11.6 years; height 1.7 ± 0.1 m; weight 71.6 ± 12.0 kg) were recruited for this non‐invasive experiment (Table 1). All provided their written informed consent to the experimental procedures, which were approved by the local human ethics committee at the University of Birmingham and conformed to the principles of the *Declaration of Helsinki*.

**Table 1:** Participant anthropometric data

Participant	Sex	Age (years)	Weight (kg)	Height (m)	Toppling torque per unit angle (Nm deg^−1^)
P01	Male	21	57.4	1.67	7.71
P02	Female	35	57.9	1.58	8.28
P03	Male	23	70.7	1.81	9.81
P04	Male	21	71.3	1.82	12.71
P05	Male	30	79.9	1.82	13.53
P06	Male	28	60.8	1.75	10.27
P07	Male	29	78.4	1.8	12.21
P08	Female	25	64.1	1.59	11.51
P09	Male	60	94.8	1.85	16.75
P10	Male	37	80.7	1.84	11.76
Mean ± SD		30.9 ± 12	71.6 ± 12	1.75 ± 0.1	11.4 ± 3

### Procedure and apparatus

**Figure 1:**
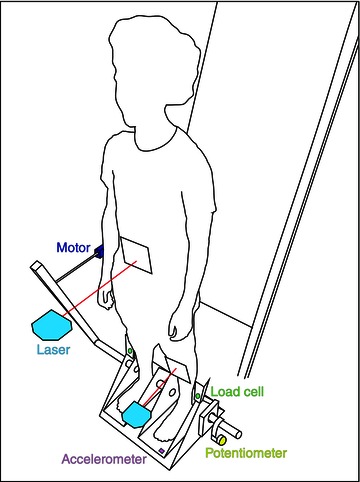
**Experimental set‐up** A horizontally‐oriented linear servo motor (situated on subject's right in picture) applied perturbations to the platform via a lever. Two load cells measured torque; a potentiometer measured anterior–posterior rotation of the footplate; an accelerometer attached underneath the left footplate measured footplate acceleration; and two laser‐reflex sensors placed at mid‐tibia and umbilicus level tracked the anterior–posterior shin and body tilt. The board seen behind the participant was adjusted to the vertical position during the board condition; the participant was strapped to it by use of polyester webbing.

**Figure 2:**
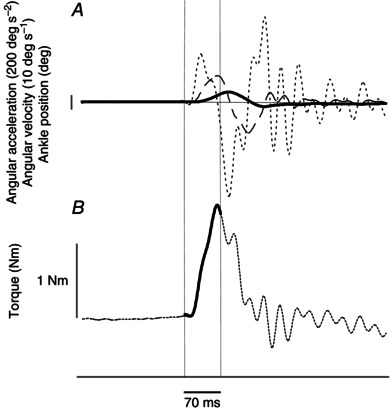
**Estimating ankle stiffness** *A*, mechanical ankle stiffness was estimated by fitting the torque response with a signal generated by a second order model that utilizes the ankle angle (continuous line), angular velocity (dashed line) and angular acceleration (dotted line) as its three inputs. The thin vertical lines indicate the time window used for the analysis (70 ms), with the starting point coincident with the stimulus onset. The horizontal line depicts zero for position, velocity and acceleration. *B*, ankle torque response (dotted line) and reconstructed torque (continuous line) obtained from the model. The horizontal line depicts 14.5 Nm.

Ankle stiffness was measured with a custom‐built footplate apparatus (Fig. 1). This consisted of a motorized platform supporting two freely moving footplates which were subjected to a common rotation. A linear motor (Model XTA3810S; Copley Motion Systems LLC, Basildon, UK) was used to rotate the platform via a lever. It operated in position‐servo mode; hence, the footplates were driven to specified positions irrespective of any resistance offered by the subject. The footplate axis was positioned 8.6 cm high to coincide approximately with the human average ankle joint height. Participants stood with each foot on separate plates and with the centre of the ankle joint aligned with the footplate axis in the sagittal direction. Small perturbations were applied with a variable gap of 4–5 s during trials of standing, which lasted for ∼3 min. Between each trial, subjects were given ∼1 min of rest, when movement was allowed. The perturbation consisted of a 7 Hz squared sine wave. This evoked a rotation of the ankle as depicted by the solid line in Fig. 2 *A*. Because ankle stiffness has previously been shown to depend on stimulus amplitude (Kearney & Hunter, 1982; Hufschmidt & Schwaller, 1987; Loram *et al*. 2007 *a*), we applied four different rotation sizes of 0.1, 0.2, 0.4 and 0.6 deg, intended to span the range of the muscle short‐range elastic component (Hunter & Kearney, 1982; Mirbagheri *et al*. 2000; Loram & Lakie, 2002; Casadio *et al*. 2005). The smallest perturbation (0.1 deg) was determined by the capability of our apparatus. Stimulus amplitude and direction (toes‐up or toes‐down) were randomized. Each subject was tested within a single session of ∼2 h, including set‐up time and breaks.

**Figure 3:**
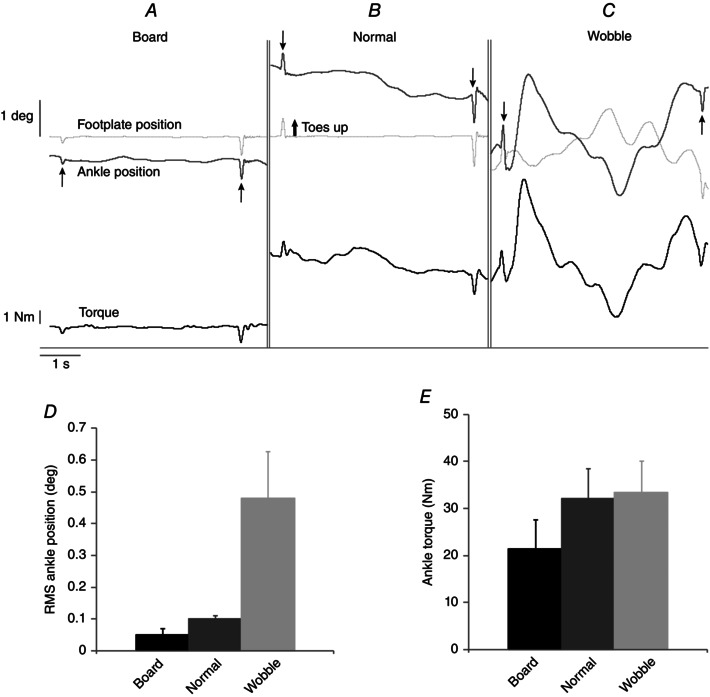
**Effect of sway condition upon ankle angle and torque** Segment of the footplate position, ankle position and torque during board (*A*), normal (*B*) and wobble (*C*) conditions. The perturbations were randomized in amplitude, direction (toes up and toes down) and interval (4–5 s). The long horizontal line spanning the bottom of (*A*) to (*C*) represents 9 Nm for all torque traces. Root‐mean‐square ankle position during a 2 s pre‐stimulus time window is shown in (*D*). Mean ankle torque during a 70 ms pre‐stimulus time window is shown in (*E*).

To determine how baseline motion of the ankle joint would affect its stiffness we artificially manipulated the degree of ankle movement in three ways:

1. Normal: participants were standing freely.
2. Board: participants were strapped to a fixed vertical body support, minimizing body (and therefore ankle) movement.
3. Wobble: participants were standing freely at the same time as the footplates were continuously rotated by a randomly‐varying waveform, generated by applying a 1 Hz low‐pass filter to white noise. The root‐mean‐square amplitude of the waveform was 0.6 ± 0.02 deg (mean ± SD). This was sufficient to increase ankle movement without endangering balance, and no subject found this condition to be challenging in the least. The stiffness measuring perturbations were summed with this waveform (Fig. 3 *C*).

The three conditions of baseline ankle movement (normal, board, wobble) combined with the four different perturbation sizes (0.1, 0.2, 0.4, 0.6 deg) resulted in a total of 12 conditions. Forty‐eight perturbations were applied per condition, resulting in a total of 576 for each participant.

Platform angular displacement, velocity and acceleration, along with ankle torque, were used to estimate stiffness of the ankle joint. Torque was measured by two miniature load cells (Model 31; Sensotec Inc., Columbus, OH, USA). These were horizontally mounted between the platform and footplates and placed directly above their axis of rotation. The foot was firmly placed on the footplate, and angular displacement was recorded with a precision Hall effect potentiometer (Model CP‐2UT; Midori Precisions Co., Tokyo, Japan) located on the platform axis. A laser‐reflex sensor (Model YT25MGV80; Wenglor, Tettnang, Germany), placed at the left mid‐tibia level (150–250 mm away from the shin), was used to record shin linear displacement, which was converted to angular rotation by taking the inverse tangent of laser distance over height. This signal was subtracted from the footplate angle to provide the ankle angle. A second laser (Model YT44MGV80; Wenglor) was used to record an approximation of the centre of mass (COM) angular position and was directed at the umbilicus. This laser was used for the estimation of the gravitational toppling torque (see below). A 3 g linear accelerometer (Model ADXL335; Analog Devices Inc., Norwood, MA, USA) measured footplate acceleration. This was attached underneath the left footplate at a distance of 0.22 m from its axis, and the signal was converted to angular acceleration. Signals were low‐pass filtered by a fourth‐order Butterworth filter with a cut‐off frequency at 40 Hz. Velocity was calculated by integrating angular acceleration. Muscle activity evoked by the perturbation (e.g. stretch reflexes) could affect our calculations of joint stiffness (Mirbagheri *et al*. 2000; Loram & Lakie, 2002; Casadio *et al*. 2005). However, using a set‐up similar to that employed in the present study, Loram & Lakie (2002) showed that such reflexes occurred well outside the 70 ms time window in which our analysis was restricted. Nevertheless, to exclude the possibility of neural intervention, we recorded surface electromyogram (EMG) activity from the tibialis anterior and lateral gastrocnemius muscles in both legs (Model Bagnoli‐8; Delsys Inc., Natick, MA, USA, bandpass filtered between 20–450 Hz). Although we did not record directly over the soleus muscle, previous research reports considerable cross‐talk between the triceps surae muscles when using surface EMG (Toft *et al*. 1991).

### Data analysis

#### Determination of mechanical intrinsic ankle stiffness

We assumed that the ankle joint acted as a rotating mass‐spring‐damper system (Agarwal & Gottlieb, 1977; Hunter & Kearney, 1982). The calf muscles (contractile element) and the tendon, aponeurosis and foot (series elastic element) act as the mass‐spring‐damper system responsible for generating the corrective torque applied by the feet against the ground to stabilize position (Fitzpatrick *et al*. 1992; Winter *et al*. 1998). The moment of inertia of the foot and moving muscle with respect to the medial malleolus acting as the axis of rotation comprises the mass component. The spring component is the combination of the muscles, tendon, aponeurosis and foot controlling stiffness of the ankles. Finally, the damper component comprises the viscosity of the joint, muscles and associated tissues. Stiffness, viscosity and moment of inertia were estimated with a fitting equation in which the torque measured over the first 70 ms of the perturbation was compared with the torque generated by a simple second‐order model. The three inputs to this model were the measured ankle position, velocity and acceleration (Fig. 2) (Kearney & Hunter, 1982; Loram & Lakie, 2002):

$$T\;=\;K\theta \;+\;B\dot{\theta }\;+\;l\ddot{\theta }$$

Where: Т is torque (Nm); θ is angle (deg); $\dot{\theta }$ is angular velocity (deg s^−1^) and $\ddot{\theta }$ is angular acceleration (deg s^−2^); *K* is stiffness (Nm deg^−1^); *B* is viscosity (Nm s deg^−1^) and *I* is moment of inertia of the foot (kg m^2^).

The reliability of the estimation process was monitored by correlating the estimated torque with the actual torque (mean *r* ^2^ = 0.99; *P* < 0.001).

#### Determination of toppling torque per unit angle, baseline ankle sway and torque

During quiet standing, body sway amplitude is below 6 deg (Hellebrandt & Braun, 1939); thus, the gravitational torque exerted by the body COM is almost perfectly related to the COM rotation around the ankle joint (Smith, 1957; Gurfinkel & Osevets, 1972; Fitzpatrick *et al*. 1992). This relationship determines the toppling torque per unit angle, and represents the minimal ankle stiffness required to stabilize the body at the vertical equilibrium point (Gurfinkel & Osevets, 1972). It can be defined as *m* × *g* × *h*, where *m* is the participant mass above the ankles, *g* is the gravitational acceleration and *h* is the height of the COM above the ankles. Although this concept is based on the body inverted pendulum model and may underestimate the relationship between changes in body COM and other joints (Aramaki *et al*. 2001; Pinter *et al*. 2008), it is used here as a reference to normalize data from all participants, regardless of their body mass and height. For passive ankle stiffness to stabilize the body alone, it must be equal to or greater than *m* × *g* × *h*. We therefore determined toppling torque per unit angle for each subject so that we could express ankle stiffness as a percentage of this value. Because we did not have precise knowledge of the height of the COM, *m* × *g* × *h* could not be calculated directly. We therefore determined toppling torque empirically as the slope of a linear fit between ankle torque (from the load cell data) and body angle (umbilical laser‐reflex sensor data). For convenience, we refer to toppling torque as ‘mgh’. Torque and body angle were recorded during 30 s of voluntary sway, when subjects were instructed to sway very gently about the ankle joint, minimizing any hip or knee motion. As expected, this value correlated strongly with subject mass (*r* ^2^ = 0.96, *P* < 0.001) and height (*r* ^2^ = 0.89, *P* = 0.001).

Our primary aim was to determine how prior ankle movement affects ankle stiffness in standing. Ankle movement was quantified as the root‐mean‐square ankle position over a two second time window prior to the onset of each stiffness‐measuring perturbation. Previous research also shows that ankle joint stiffness increases as a function of ankle torque (Hunter & Kearney, 1982; Casadio *et al*. 2005). Therefore, we also measured mean ankle torque during a 70 ms time window immediately prior to each perturbation.

#### Statistical analysis

Repeated‐measures ANOVA was used to determine effects of condition (wobble, normal, board) and stimulus amplitude (0.1, 0.2, 0.4, 0.6 deg) upon ankle stiffness. Pearson's correlation was used to investigate the relationship between baseline ankle torque and stiffness. P < 0.05 was considered statistically significant for all tests.

## Results

### Ankle movement

Figure 3 shows representative data for all three conditions: board (Fig. 3 *A*), normal (Fig. 3 *B*) and wobble (Fig. 3 *C*). Within the 5.4 s period shown for each condition, two ankle perturbations can be identified. The traces illustrate the wide range of spontaneous ankle movement and torque observed across conditions; the average baseline results are summarized in the bar graphs (Fig. 3 *D* and *E*). Although footplate rotation induced by the perturbation was identical between board and normal, baseline ankle movement and torque was greater for the latter. The minor fluctuations that occurred in the board condition represented the limitations of our ability to immobilize the subject. During the wobble condition, when a randomized waveform was applied to the footplates, ankle motion was inevitably much greater, as was the intention. Mean pre‐stimulus ankle movement exhibited a significant difference between conditions, approximately doubling in value between board and normal, with a much larger increase again for wobble (*F*_2,18_ = 82.5; *P* < 0.001) (Fig. 3 *D*). This confirmed that our interventions were successful in manipulating the degree of baseline ankle motion prior to each stiffness‐measuring perturbation.

### Ankle stiffness, viscosity and inertia

**Figure 4:**
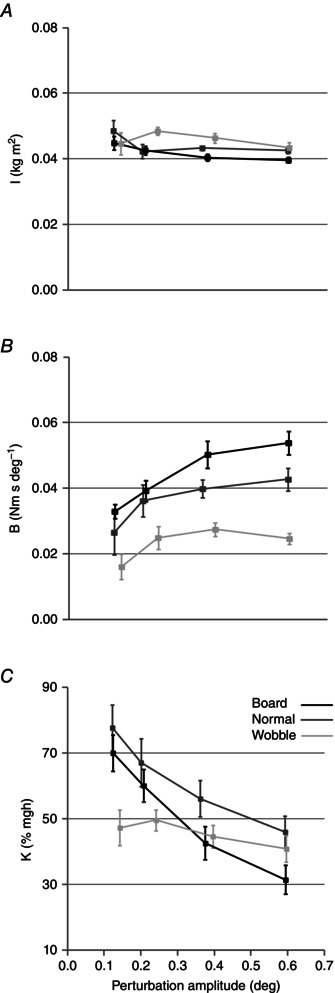
**Ankle stiffness, viscosity and inertia for all conditions and perturbation amplitudes** *A*, ankle inertia (*I*). *B*, viscosity (*B*). *C*, stiffness (*K*) (mean ± SEM). Values of inertia and viscosity are for one ankle only. Stiffness values have been multiplied by two to account for both legs, and are expressed as a percentage of gravitational toppling torque (mgh).

There was no effect of perturbation direction (toes‐up *vs*. toes‐down) upon stiffness, viscosity or inertia (*F*_1,9 _< 1.1; *P* > 0.32). Both directions were therefore combined for all further analysis. Figure 4 *A–C* depicts mean ankle stiffness, viscosity and inertia for all conditions and perturbation amplitudes. The estimated inertia of the combined foot and footplate remained similar across perturbation amplitudes (*F*_3,27_ = 2.9; *P* = 0.055). However, there was a significant influence of condition upon inertia, reflecting slightly higher values with increasing baseline ankle movement (*F*_2,18_ = 3.92; *P* = 0.039). Viscosity increased with perturbation amplitude (*F*_3,27_ = 23.8; *P* < 0.001) and became larger with increasing ankle movement (condition effect: *F*_2,18_ = 25.5; *P* < 0.001). Crucially, neither inertia, nor viscosity exhibited an interaction between amplitude and condition (*P* > 0.11), in contrast to ankle stiffness, which is reported below.

Because we were primarily interested in estimating passive ankle stiffness, we first needed to exclude the possibility of an active contribution to the ankle torque as a result of the perturbation (e.g. stretch reflexes). We therefore compared mean EMG activity of the lateral gastrocnemius between 70 ms time windows pre‐ and post‐stimulus but found no significant difference (pre *vs*. post; *F*_1,9_ = 0.4; *P* = 0.54). Figure 4 *C* depicts mean ankle stiffness for all conditions and perturbation amplitudes, reported here as a percentage of toppling torque per unit angle (‘% mgh’). Values ranged between 31% and 78% mgh. For both the normal and board conditions, there was a systematic non‐linear reduction in ankle stiffness with increasing perturbation amplitude. This effect was absent for the wobble condition, where stiffness was relatively low, and remained low (31–49% mgh) across all amplitudes. These observations are confirmed by a significant interaction between condition and amplitude (*F*_6,54_ = 7.6; *P* < 0.001). This effect of wobble is consistent with our hypothesized effect of prior muscle movement upon joint stiffness. By contrast to our hypothesis, however, stiffness was slightly but significantly lower in the board compared to normal condition, across all perturbation amplitudes. We speculated that changes in baseline torque between conditions may underlie the difference.

### Ankle stiffness normalized against baseline torque

**Figure 5:**
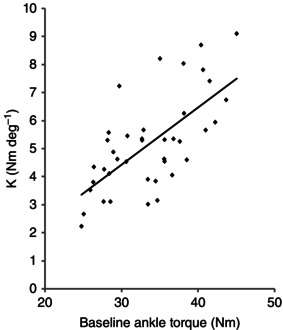
**Effect of baseline ankle torque upon stiffness** The relationship between pre‐perturbation torque and ankle stiffness is presented for all amplitudes during the wobble condition. Data from board and normal are not included because they exhibited an additional significant effect of perturbation amplitude upon stiffness.

**Figure 6:**
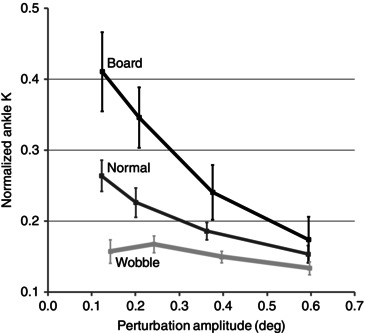
**Normalized ankle stiffness** Absolute ankle stiffness was divided by mean torque during a 70 ms pre‐stimulus time window torque to obtain normalized values (mean ± SE).

**Figure 7:**
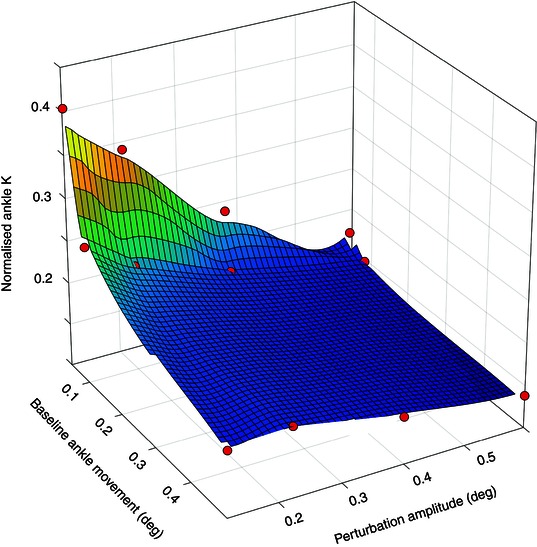
**The influence of baseline movement and perturbation amplitude upon ankle stiffness** Mean data are shown along with a cubic spline three‐dimensional interpolation.

To test this speculation, we compared baseline torque during a 70 ms pre‐stimulus window (Fig. 3 *E*). Values were almost identical between normal and wobble (∼33 Nm) but were ∼36% less for board (*F*_2,18_ = 16.4; *P* < 0.001). We then verified the correlation between torque and stiffness. We restricted this analysis to the wobble condition because it was the only one in which stiffness was not affected by perturbation amplitude. Figure 5 shows a significant positive correlation of torque (Nm) against absolute stiffness (Nm deg^−1^) (*r* ^2^ = 0.67; *P* < 0.001). We therefore normalized stiffness values by dividing them by baseline ankle torque. The result is shown in Fig. 6. After this normalization procedure, the non‐linear qualitative shape of the board and normal results remains the same, although now stiffness is highest during the condition with least ankle movement (board). Furthermore, at the highest perturbation amplitude, stiffness converges towards the same value for all conditions. A combined view of the two factors influencing ankle stiffness is shown three‐dimensionally in Fig. 7. The cubic spline interpolation shows that stiffness decreases as a function of both prior ankle movement and perturbation amplitude.

### Identifying the short‐range stiffness component

**Figure 8:**
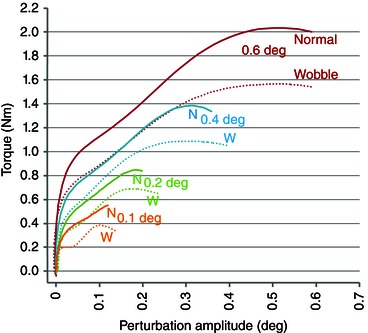
**Torque–angle relationship during each perturbation** Data are shown for normal (continuous) and wobble (dotted) conditions for all perturbation amplitudes.

To identify changes in stiffness throughout the time course of each perturbation, we examined the relationship between ankle torque and position during the first 70 ms of each stimulus. Figure 8 shows the results for normal and wobble conditions for all perturbation amplitudes (the board condition presented similar results; however, it was not included here because the baseline ankle torque was lower, precluding direct comparison). The gradient between torque and angle is a function of stiffness. An initial steep rise in torque can be seen at the onset of ankle movement for all conditions, consistent with the short‐range muscle stiffness component. This is followed by a much shallower rise in torque for the remainder of the perturbation. For all perturbation amplitudes, the transition between these two phases occurs at a lower torque and amplitude during the wobble condition.

## Discussion

A passive ankle stiffness value equating to 100% of the body's gravitational toppling torque would be sufficient to stabilize the body, assuming a sway frequency of zero. However, previous research suggests that, for empirically‐observed sway frequencies of ∼0.5 Hz, this value would need to be ∼200% to completely stabilize the body through passive means alone (Winter *et al*. 1998; Morasso *et al*. 1999; Morasso & Schieppati, 1999; Lakie *et al*. 2003). In agreement with other studies, our estimates of *K* were well below this value, ranging from between 31% and 78% mgh (91% in Loram & Lakie 2002; 64% in Casadio *et al*. 2005). This confirms that passive stiffness alone is insufficient for even minimal stabilization in standing, and suggests that active mechanisms must modulate ankle torque by changing calf muscle activity. Nevertheless, it is clear that the passive mechanism does contribute to balance, and previous results demonstrate considerable variation between people. The present study aimed to determine how it may change within a standing subject as a result of changes in ankle joint motion with baseline sway. Our results demonstrate significant changes in ankle stiffness within the same person depending upon their baseline sway. This suggests that the relative contribution of the active and passive mechanisms to balance changes over time depending on circumstances.

In addition to manipulating the level of baseline ankle sway, we also measured stiffness across a range of stimulus amplitudes. For both the normal and board conditions, there was a non‐linear reduction in *K* with increasing stimulus amplitude (78–46% and 70–31% mgh, respectively). Such amplitude‐dependence has previously been demonstrated in both the wrist and ankle joints (Halaki *et al*. 2006; Kearney & Hunter, 1982; Loram *et al*. 2007 *a*, 2007 *b*; Vlutters *et al*. 2015), and this disproportionately high passive resistance to the initial stages of imposed stretch has been attributed to the ‘short‐range stiffness’ of muscle tissue (Rack & Westbury, 1974). Taking into account this amplitude‐dependence, the range of *K* that we observed in the board and normal conditions is in good agreement with previous findings. Although, when measuring *K* in individuals attached to a board, Loram & Lakie (2002) observed a *K* larger than the largest estimate seen in the present study (91% *vs*. 70%), their perturbation was around half the magnitude of our smallest perturbation (0.055 *vs*. 0.1 deg). The results from Loram *et al*. (2007*a*), also obtained with participants attached to a board, are more similar, especially for short slow stretches. Even though Loram *et al*. (2007*a*) used repetitive contiguous triangular‐shaped stimuli, which potentially diminished the thixotropic effect on stiffness, they predicted values of 67–54% for 0.15–0.4 deg perturbations, which are very similar to our estimates of 70%, 60% and 42% for 0.1, 0.2 and 0.4 deg perturbations. When calculating *K* using long slow continuous stretches, they estimated 30–40% for 1 deg perturbations, as opposed to our estimates of 31% for 0.6 deg. In their study, the stimulus velocity was much lower than the velocity used here (∼0.35 *vs*. 5–22 deg s^–1^), suggesting that amplitude is the key stimulus property affecting estimates of *K*.

The main objective of the present study was to determine whether ankle stiffness is altered by the magnitude of baseline sway around the ankle joint. We hypothesized that *K* would be inversely related to ankle sway. This hypothesis is based upon the well‐established thixotropic property of muscle tissue (Buchthal & Kaiser, 1951; Lakie *et al*. 1984; Hufschmidt & Schwaller, 1987; Proske *et al*. 1993; Campbell & Lakie, 1998; Proske & Morgan, 1999; Axelson & Hagbarth, 2001; Whitehead *et al*. 2001; Reynolds & Lakie, 2010). Specifically, it has been shown that short‐range stiffness is significantly reduced following muscle movement but progressively recovers if muscle movement is minimized for some time. Hence, the immediate history of calf muscle motion would also be expected to influence overall standing ankle stiffness. In the present study, we manipulated the degree of calf muscle motion by changing ankle motion across stance conditions (board, quiet, wobble). The fast brief perturbations that we used to estimate stiffness might also be expected to affect stiffness by themselves. However, previous research suggests that the thixotropic time constant of the ankle joint (i.e. the time taken to recover most of the stiffness) is ∼4 s (Hufschmidt & Schwaller, 1987). By adopting an interstimulus interval of 4–5 s, we therefore allowed sufficient time for the ankle musculature to recover the majority of its resting stiffness between perturbations. More importantly, the interstimulus interval was identical between the three stance conditions. Root‐mean‐square ankle movement became progressively larger from board to normal to wobble, confirming that our interventions were successful in manipulating baseline ankle sway. In confirmation of our hypothesis, the condition with the highest degree of ankle motion (wobble) exhibited the lowest stiffness, being 41–49% for all perturbation amplitudes. Board and normal exhibited higher stiffness, although this was only apparent at the lowest stimulus amplitude. As the amplitude increased, stiffness values tended to converge towards a low value for all three conditions. This caused a statistical interaction between condition and stimulus amplitude, which can be explained by taking into account the short‐range stiffness of muscle described above. At the largest perturbation amplitude, the muscle is stretched beyond its short‐range threshold, becoming much less stiff. The large perturbation will therefore tend to be dominated by the lowest stiffness that the muscle can achieve, producing a floor effect for all conditions. This agrees with the findings of Loram *et al*. (2007*a*), who used ultrasound to track the origin of stiffness changes with increasing amplitude. With small perturbations, they observed minimal muscle movement for a given ankle rotation. As the amplitude increased, muscle movement became disproportionately larger. Loram *et al*. (2007*a*) concluded that small perturbations mostly stretch the Achilles tendon because the muscle is much stiffer. As the amplitude increases, the muscle is stretched beyond the short‐range stiffness, producing a profound fall in overall ankle stiffness and a greater degree of muscle movement. Figure 8 comprises a visual representation of this phenomenon. The gradient between torque and angle varies as a function of stiffness, and the time period is the same as used for the stiffness estimation procedure (70 ms). In all conditions, the most prominent change in the steepness of the slope, which is present at a very early phase, marks the transition between short and long range stiffness. The overall stiffness is a composite of these two phases. During the initial phase, the muscle moves less and the tendon stretches most. Torque then rises less rapidly as the muscle is stretched beyond this point and the stiffness of the contractile component is dramatically reduced. The greater the proportion of the initial steep rise in the overall torque curve, the higher the overall stiffness of the ankle joint. For all perturbation amplitudes, this initial steep rise is consistently lower in wobble compared to normal condition, showing that the relatively low stiffness found in the wobble condition can be related to reduction in the range of the short‐range stiffness. Furthermore, for small amplitudes, the initial rise in stiffness is proportionally more representative of the overall stiffness. This explains the higher stiffness values found when the ankle is moved by a small amount.

The results did not completely agree with our hypothesis, at least initially. We expected to see higher stiffness for board compared to normal but saw the opposite across all amplitudes. This raises the issue of an additional parameter known to affect ankle stiffness, namely torque. As the muscle generates progressively more torque, more cross‐bridges form, increasing muscle stiffness and the resistance to an imposed perturbation. Hence, estimates of stiffness will depend upon the contractile state of the muscle. This was demonstrated by Hunter and Kearney (1982) who found that the non‐linearities of ankle stiffness were dependent not only upon displacement amplitude, but also on variations of ankle torque. We confirmed this effect in our data (Fig. 4) and, furthermore, found a significant difference in baseline ankle torque between conditions. The board condition exhibited ∼36% less torque than normal and wobble conditions, which were similar to each other (mean ± SD: 21.5 ± 7.5 Nm *vs*. 33.4 ± 6.7 Nm and 33.6 ± 5.8 Nm). This would explain the consistently lower values of stiffness in board compared to normal condition, across all amplitudes. It also suggests that in leaning forward, when more torque is required, stiffness will increase, potentially increasing stability. This is a possible reason for not standing strictly at the vertical equilibrium point. However, other reasons might include minimizing the range of backward COP movement and/or involvement of the dorsal flexors. After factoring out differences in baseline torque, the data fully confirmed our hypothesis (Figs 6 and 7). The board condition exhibited the highest (normalized) stiffness, followed by normal then wobble. As stimulus amplitude increased, the difference between conditions progressively reduced, reaching a floor value at the highest amplitude (0.6 deg). Figure 5 shows that, as baseline torque increased from ∼25 to 45 Nm, stiffness increased from ∼3 to 7 Nm deg^−1^. The maximal effect of stance condition was to increase stiffness from 0.15 to 0.4 (normalized *K*) at the largest amplitude (Fig. 6). Hence, the thixotropic effect upon stiffness was considerable.

The potential consequence of reduced ankle stiffness is to increase reliance upon active neural intervention to maintain balance. This would not only involve more torque modulation, but also faster modulation. Loram *et al*. (2007*a*) explained the importance of increased passive stiffness in raising the time constant of the unstable, inverted pendulum‐like, body. An increased time constant decreases the acceleration of the toppling body and in effect ‘buys time’ for the nervous system to act. The relevant equation for the time constant (tau) is: $\tau =\sqrt{\frac{I}{mgh(1-c)}}$ where *c* is normalized stiffness, *I* is moment of inertia, m is mass, *h* is height and *g* is acceleration as a result of gravity. If the moment of inertia is written as *I* = *kmh* ^2^ where *k* is a shape factor of value ∼1.3 (Morasso & Sanguineti, 2002), the time constant becomes: $=\sqrt{\frac{kh}{(1-c)g}}$. If we assume the COM is positioned at 55% of our subjects’ height (1.75 m), this equates to an *h* of 0.96 m. During normal standing, *c* ranged from 0.46 to 0.78, at 0.6 and 0.1 deg of stimulus amplitude, respectively. Therefore, these stiffness values equate to time constants of 491 and 756 ms. This shows that the behaviour of the standing subject and the size and timing of their neural response are all sensitive to the size of the perturbations applied. During the wobble condition, the time constant was always ∼490 ms. This suggests that greater control alacrity is required in situations where sway size is large, such as when standing in moving vehicles.

The results reported in the present study suggest that individuals with less stiffness may be less stable. To our knowledge, only Fitzpatrick *et al*. (1992) has investigated this by showing that, in individuals who were instructed to stand at ease, physical perturbations produced larger disturbances than in those attempting to stand still. However, Fitzpatrick *et al*. (1992) did not report the size of the spontaneous sway in the two conditions and did not measure intrinsic ankle stiffness. The present study does both, and shows that the intrinsic stiffness is less in individuals who are (or have recently been) swaying more. Whether these stiffness changes have consequences for larger perturbations or affect postural stability in the widest sense remains to be seen.

For an ankle stiffness dependent on the history of movement, the implication is that the demand for neural intervention to stabilize standing will not be constant but, instead, will vary continuously. For control of limb movement, a reduction in stiffness as movement occurs is favourable because it allows muscles to economically control both posture and movement. For standing, it may be less beneficial because an increased sway will lead to a reduction in ankle stiffness and stability and thus, potentially, to collapse unless there is additional neural intervention.

## Additional information

### Competing interests

The authors declare that they have no competing interests.

### Author contributions

TS, ML and RR contributed to all aspects of the work, including the conception of the experiments, the experimental design and collection and assembly of data, analysis and interpretation of data, drafting of the article, and critical revision for important intellectual content. All authors approved the final version of the manuscript, all persons designated as authors qualify for authorship, and all those who qualify for authorship are listed.

### Funding

This work was supported by the University of Birmingham Brazil Scholarship for Research Excellence and BBSRC grant BB/L02103X/1.
